# Analysis of the Contribution of Petroleum Acid Components to the Viscosity of Heavy Oils with High TAN

**DOI:** 10.1021/acsomega.3c04098

**Published:** 2023-07-26

**Authors:** Shuang Liu, Jianxun Wu, Zhiming Xu, Linzhou Zhang, Suoqi Zhao

**Affiliations:** State Key Laboratory of Heavy Oil Processing, China University of Petroleum, Beijing 102249, P. R. China

## Abstract

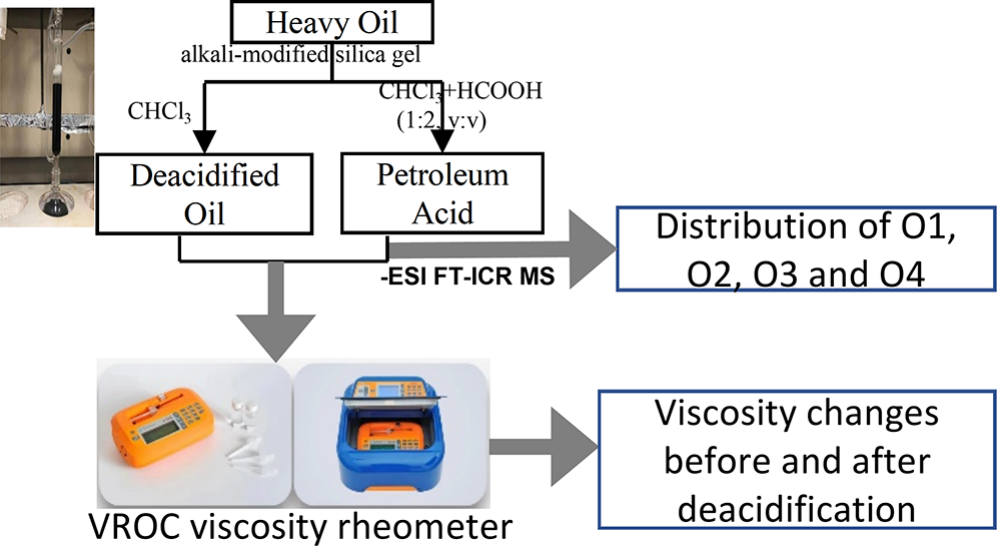

The viscosity of heavy oil hinders its cold production, posing a major challenge to its exploitation. The high viscosity of heavy oil can be attributed to the content of asphaltene. However, during the collection of heavy oil samples from various regions in China, we observed that heavy oils with high total acid number (TAN) but low asphaltene content also exhibit relatively high viscosity. Hence, the viscosity mechanism of high-acid crude oil, the influence of petroleum acid on heavy oil viscosity, should be investigated. In this study, Xinjiang Chunfeng heavy oil was selected for analysis, possessing a viscosity of 16,886 mPa·s at 50 °C and a high total acid number (TAN) of 17.72 mg KOH/g. Separation was performed on the deacidified oil and the acid component using an alkali-modified silica gel column. The viscosity changes of the deacidified oil and its blends with varying proportions of the acid component were determined, along with the viscosity changes of the deacidified oil and acid components in a toluene solution. The molecular composition was analyzed using a Fourier transform ion cyclotron resonance mass spectrometer (FT-ICR MS). The findings indicated successful separation of petroleum acid from the heavy oil, the acid component yield being 16.65 wt %. Furthermore, the viscosity of the petroleum acid was significantly higher than that of the deacidified oil. The rate of viscosity change of the acid component in the toluene solvent exceeded that of the deacidified oil, and the viscosity of the deacidified oil notably increased upon the addition of acid. In conjunction with the viscosity data, it was observed that the deacidified oil exhibited the removal of O2 and O4 compounds, resulting in a 43.11% viscosity reduction at 30 °C compared with crude oil. Thus, the monoacid and diacid components considerably affected the viscosity of heavy oil.

## 1 Introduction

The depletion of conventional light and medium oil resources has led to increased attention on effectively and economically exploiting vast unconventional heavy oil and bitumen resources.^1^ Heavy oil is an essential resource that plays an increasingly prominent role in the global oil supply.^2−5^ However, the high viscosity and poor fluidity of heavy oil present significant challenges to its recovery.^6−8^ Heavy oil exhibits complex compositions with varying properties across different regions. The viscosity of heavy oil is comprehensively influenced by factors such as asphaltene and resin content, as well as elemental compositions. The substantial viscosity of heavy oil severely hampers the development of its mining, gathering, and transportation processes. Therefore, investigating the key factors that contribute to heavy oil viscosity is crucial to address this challenge and provide theoretical guidance for subsequent mining, transportation, and viscosity reduction efforts.

In previous studies, asphaltene content has been widely acknowledged by scholars as a crucial factor in determining the high viscosity of heavy oil.^9−12^ Experimental investigations have demonstrated a close correlation between the viscosity of heavy oil and the volume fraction, chemical structure, and physicochemical properties of asphaltene, which is often the most polar and heaviest component in heavy oil.^13,14^ The influence of asphaltene content on heavy oil viscosity has been extensively explored. Mack^15^ conducted viscosity measurements on Mexican bitumen, clearly illustrating that viscosity increases with increasing asphaltene content. At room temperature, the viscosity of a blended oil containing 20 vol % asphaltene is 367 times higher than that of deasphalted oil (maltenes). This substantial increase in viscosity is attributed to the strong particle aggregation of asphaltene. Dealy^16^ introduced 5 wt % asphaltene to an Athabasca bitumen sample with an initial bitumen content of 16 wt % (*n*-butane insoluble) and observed an increase in bitumen viscosity from 300,000 to 1,000,000 mPa s. Luo et al^1^ attributed the high viscosity of heavy oil to the leading role played by asphaltene content in heavy oil samples. They observed that when the asphaltene content is high, the strong, attractive interactions between asphaltene particles result in a sharp increase in heavy oil viscosity. Mahdi^9^ experimentally demonstrated that under constant temperature conditions, the viscosity of compound heavy oil exponentially increases with increasing asphaltene content. Crude oil viscosity primarily relates to the composition, chemical structure, and asphaltene content of the crude oil,^17^ with asphaltene considered the heaviest and most polar component in crude oil.^18^ The asphaltene content in crude oil is widely regarded as a reliable indicator of both crude oil and asphalt viscosity.^19,20^ Crude oils with higher asphaltene content typically exhibit stronger viscosity, which can be attributed to the stronger attraction and aggregation of asphaltene particles, especially in heavy or superheavy crude oils. Asphaltene content serves as a highly sensitive parameter for crude oil and asphalt viscosity.

While high asphaltene content is associated with high viscosity, few studies have focused on crude oil with low asphaltene content (<2%). We discovered that Xinjiang cold heavy oil exhibits high viscosity despite its low asphaltene content. Therefore, the main factors contributing to the viscosity of this particular crude oil type should be investigated. Petroleum acid is a natural surfactant,^21^ which also has a certain effect on viscosity and rheological properties. Petroleum acids mainly refer to naphthenic acids (about 95%), fatty acids, aromatic acids, and other acidic substances in crude oil. The acidic material of the oil sample contains high contents of N, S, and O heteroatoms, and a large number of ring structures occupy a large proportion. Naphthenic acids are the cause of the formation of emulsions in crude oil during production and are also the cause of metal corrosion caused by organic acids during transportation and refining. The assessment of NA content in petroleum and its products can be given in terms of total acid number (TAN), which is defined as the amount in milligrams of potassium hydroxide needed to neutralize 1 g of oil sample. Naphthenic acids (NAs) are structured with acidic oxygenated functionalities, and their chemical formula is represented as C*_n_*H_2*n*+z_O_2_.^22−24^ Wanfen Pu^25^ confirmed that the presence of petroleum acid does not favor oil–water emulsification. Although petroleum acid possesses an amphiphilic structure as a surface-active substance, the composition of naphthenic acid, fatty acid, and aromatic acid within petroleum acid can influence its hydrophilicity and lipophilicity. Naphthoic acid was easily converted to sodium naphthoate (NaNs) in the presence of NaOH. It has been reported that when asphaltenes are precipitated with n-pentane, these NaNs are still mainly dissolved in maltenes.^26^ While numerous studies have investigated the effects of natural surfactant-petroleum acid on oil–water emulsification, the effect of petroleum acid on viscosity remains largely unexplored. Therefore, our research aims to investigate the influence of petroleum acid on the viscosity of heavy oil.

The commonly employed methods for separating petroleum acids include acid–base extraction,^27,28^ solid-phase extraction,^29^ and ionic liquid extraction.^30,31^ However, acid–base extraction and ionic liquid extraction tend to yield low amounts of acids. Therefore, we utilized a solid-phase extraction method known as the alkali-modified silica gel column method to separate petroleum acids^32^ and made some improvements. FT-ICR MS provides ultrahigh mass resolution power and mass accuracy that result in a high degree of confidence in the molecular weight assignments, consequently being suitable for the analysis of complex mixtures in petroleomics. The rapid advances in mass spectrometry and related technologies in the last two decades have revolutionized our understanding of heavy petroleum composition.^33^ For example, FT-ICR MS, associated with negative-ion-mode electrospray ionization (ESI(−)), is often used for the analysis of polar compounds.^34^ Maowen Li et al.^35^ using FT-ICR MS found that the acidic component of non-biodegradable low-TAN oil was mainly composed of *n*-fatty acids, with few cyclic components. In contrast, monocyclic, bicyclic, and tricyclic acids were found to be more abundant relative to acyclic carboxylic acids among the cycloalkynes of the O2 class compounds in the high-acid oil. Subsequently, the acidic compounds were directly characterized using ESI and Fourier transform ion cyclotron resonance mass spectrometry (FT-ICR MS) to investigate the impact of petroleum acid removal on the viscosity of high naphthenic heavy oil.

In this study, the major purposes were to investigate the influence of acid components on the viscosity of heavy oil with low asphaltene content. We focused on the high-acid heavy oil found in the Xinjiang Chunfeng Oilfield. Following dehydration and other necessary pretreatments, the petroleum acid component in the heavy oil was separated using the alkali-modified silica gel Soxhlet extraction technique. The viscosity changes of the deacidified oil and its blends with varying proportions of the acid component were determined, along with the viscosity changes of the deacidified oil and acid components in a toluene solution. The properties of the heavy oil, its deacidified oil, and the acid component were analyzed, encompassing elemental compositions, molecular weight and distribution, SARA components, and TAN, among others. The separated nonacid and acid components were then analyzed using ESI high-resolution mass spectrometry, enabling the characterization of the molecular compositions.

## 2 Experimental Section

### 2.1 Materials

The reagents used in this study were analytically pure dichloromethane, *n*-hexane, formic acid, chloroform, and petroleum ether. All solvents employed in the experiments were distilled individually. Isopropanol of HPLC grade, analytically pure potassium hydroxide, and ultrapure water were also utilized.

The heavy oil sample used in this research is the CHUNFENG HEAVY OIL sample from the Chunfeng Oilfield in Xinjiang, China. According to the GBT7304-2000 method, the acid value of CHUNFENG HEAVY OIL is 17.72 mg KOH/g, classifying it as high-acid heavy oil.

### 2.2 Geological Background of Chunfeng Heavy Oil

According to the study of Liu et al.,^36,37^ Xinjiang is rich in heavy oil resources, and the Lukeqin structural belt in Turpan-Hami Basin of Xinjiang is the main heavy oil accumulation zone. The formation and distribution of heavy oil in the Lukeqin structure is controlled by the northern Permian hydrocarbon source kitchen. Its main oil generation occurred in the Late Triassic–Early Jurassic. Xinjiang Chunfeng heavy oil in this study is located in the Chepaizi gentle slope belt in the western margin of Junggar Basin. The Chepaizi convex heavy oil has the characteristics of Permian crude oil generation, mainly from the Permian source rock in Changji Depression, and the mixed source of Jurassic source rock in the later stage.^38^ These heavy oils in Xinjiang come from severe washing or severe biodegradation. Biodegradation damages saturated and aromatic hydrocarbons in crude oil to varying degrees, increases the relative contents of non-hydrocarbon components and asphaltenes, and thickens the crude oil gradually. Xinjiang Chunfeng heavy oil in our study also belongs to heavy oil with heavy biodegradation, and biodegradation is the main factor causing high acidity of crude oil. Therefore, Xinjiang Chunfeng heavy oil has poor fluidity and high acid value after severe biodegradation, so there are relatively more O1, O2, and O4 compounds in Xinjiang Chunfeng heavy oil.

### 2.3 Experimental Installation

To ensure repeatability, the properties of the raw materials were measured at least twice. Viscosity measurements were conducted using a RotoVisco 1 Rheometer TCL/2376-0010 rotary viscometer. Density was determined via the pycnometer method. Molecular weight and its distribution were measured using GPC. Family composition analysis was performed using the SARA standard program. Elemental analysis (CHONS) was conducted using the PerkinElmer CHNS/O Analyzer 2400 (PerkinElmer Co).

The molecular composition analysis was carried out using a Bruker’s Apex Ultra 9.4 T Fourier transform ion cyclotron resonance mass spectrometer (FT-ICR MS). The main parameters used were as follows: ESI was employed as the ionization source, the negative-ion mode was utilized, and the data acquisition range was set between 200 and 900 Da.

### 2.4 Experimental Method

Given the water content in the heavy oil obtained from the Chunfeng Oilfield in Xinjiang, dehydration of Xinjiang Chunfeng heavy oil was performed as the initial step. The specific procedure involved taking 10 g of Xinjiang Chunfeng heavy oil sample, adding 100 mL of petroleum ether, and conducting azeotropic distillation dehydration using a heating sleeve and water separator.

**Figure 1:**
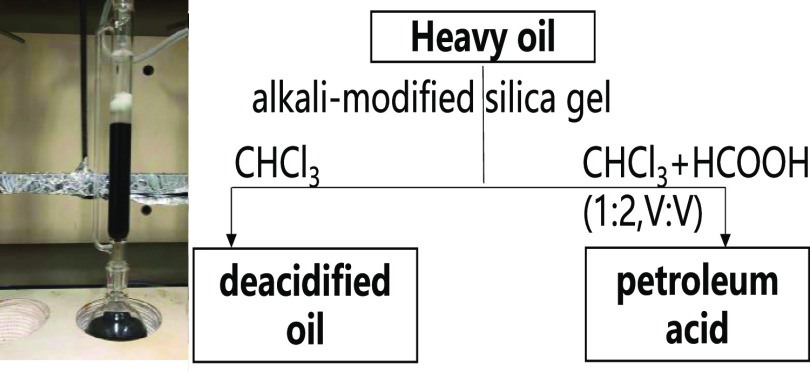
Operation flow chart of soxhlet extraction and separation of alkali-modified silica gel.

Following dehydration, the Chunfeng heavy oil was subjected to petroleum acid separation. The experiment employed the alkali-modified silica gel extraction column method with certain improvements. The procedure was as follows: initially, 100–200 mesh silica gel was extracted and modified with an alkaline KOH solution, after which the modified silica gel was packed into the extraction column. Then, ∼1 g of Xinjiang Chunfeng sample was weighed, dissolved in chloroform, and loaded onto the column. Subsequently, 2 g of silica gel and a cotton mass were added to the top of the extraction column to prevent backflow. The nonacid component was extracted using ∼150 mL of CHCl3 for 8 h, while the acid component was extracted using ∼150 mL of a mixed solvent (2:1, v/v) consisting of CHCl_3_ and HCOOH. The specific process is illustrated in Figure 1.

## 3 Results and Discussion

### 3.1 Viscosity Comparison of Heavy Oil with Different Asphaltene Contents

**Figure 2:**
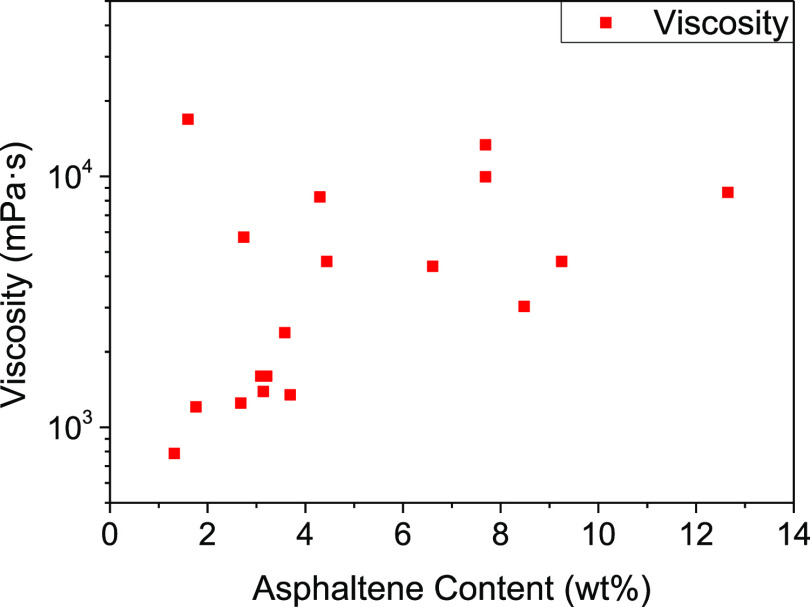
Asphaltene content–viscosity variation diagram of different oil samples.

Heavy oil refers to crude oil with a viscosity ranging from 50 mPa·s (gas-bearing crude oil) to 10,000 mPa·s (degassed crude oil) under an oil reservoir temperature and a density at 20 °C greater than 0.932 or API less than 20. In this study, we selected heavy oils from various regions in China and measured their four-component content, viscosity, and acid value. Figure 2 illustrates that the majority of oil samples from the Xinjiang Chunfeng Oilfield exhibit low asphaltene content, high viscosity, and high acid value.

Heavy oil with low asphaltene content may also exhibit high viscosity. Therefore, to investigate the viscosity of low asphaltene heavy oil, we specifically selected Xinjiang 4# heavy oil, which has an asphaltene content of 1.87 wt % and a viscosity of 16,886 mPa·s (referred to as Chunfeng heavy oil hereafter).

### 3.2 Analysis of the Basic Properties of Chunfeng Heavy Oil in Xinjiang

Analysis of the basic properties revealed that the oil samples obtained from the Xinjiang Chunfeng Oilfield belong to heavy oil characterized by low asphaltene content and high acid content, resulting in high viscosity. In order to explore the viscosity mechanism of this type of high-acid heavy oil, we utilized Xinjiang Chunfeng heavy oil as the standard sample (the total acid number, TAN, is 17.72 mg KOH/g). The basic properties of Chunfeng heavy oil are presented in Table 1.

**Table 1:** Properties of Chunfeng Heavy Oil

property	Chunfeng heavy oil
density at 20 °C, g/cm^3^	0.984
viscosity at 50 °C, mPa·s	16886
TAN, mg KOH/g	17.72
molecular weight	619
MW/Mn	5.55
C, wt %	86.08
H, wt %	9.16
H/C	1.28
N, wt %	0.59
S, wt %	0.21
O, wt %	2.50
Ca, mg/Kg	739.7
Fe, mg/Kg	31.1
Na, mg/Kg	807.3
Ni, mg/Kg	5.3
V, mg/Kg	0.1
saturates, wt %	44.43
aromatics, wt %	30.91
resins, wt %	22.79
asphaltenes, wt %	1.87

### 3.3 Analysis of Viscosity and Basic Properties before and after Deacidification

#### 3.3.1 Changes in the Acid Values and Other Properties after Deacidification

**Figure 3:**
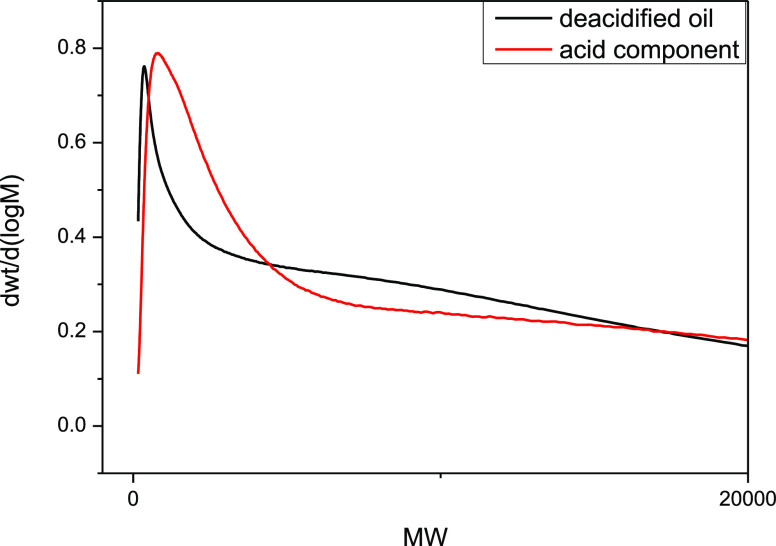
Molecular weight distribution of deacidified oil and acid component.

Initially, the total acid number (TAN) of Chunfeng heavy oil was measured both before and after deacidification. As presented in Table 2 and Figure 3, a significant reduction in acid value was observed following deacidification. The TAN decreased to 2.05 mg KOH/g, indicating an 88.43% deacidification rate. These findings demonstrate the effectiveness of the alkali-modified silica gel column method in removing a substantial portion of the petroleum acid components from the high-acid heavy oil. The elemental analysis presented in Table 2 further supports this outcome, revealing an oxygen content of 0.6% in the deacidified oil compared to the 5.59% oxygen content in the acid component. This suggests that the deacidification process enriches the acid component with petroleum acid.

**Table 2:** Changes in Acid Value and SARA Components before and after Deacidification

property	heavy oil	deacidified oil	acid component
recovery rate, wt %		75.43	16.65
TAN, mg KOH/g	17.72	2.05	
saturates, wt %	44.43	48.84	
aromatics, wt %	30.91	28.07	
resins, wt %	22.79	21.67	
asphaltenes, wt %	1.87	1.43	
C, wt %	86.08	87.03	81.18
H, wt %	9.16	11.56	11.37
H/C	1.28	1.59	1.68
O, wt %	2.50	0.60	5.59
N, wt %	0.59	0.25	0.31
S, wt %	0.21	0.23	0.28
Mn	619	619	876
*f*_A_		0.23	0.19
*f*_N_		0.25	0.18
*f*_P_		0.52	0.64

**Figure 4:**
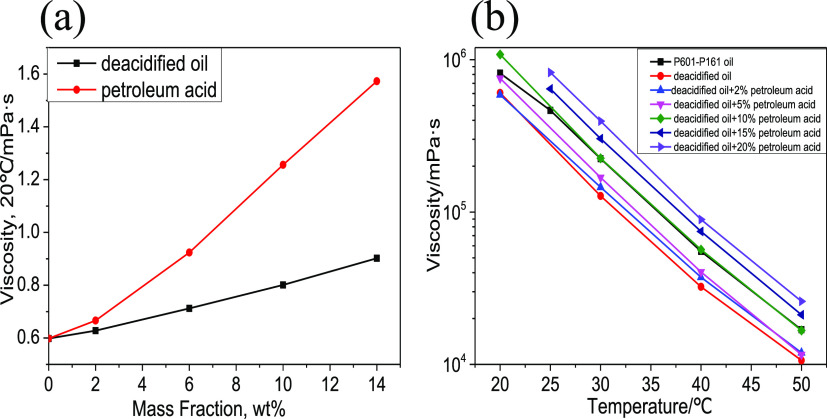
Viscosity characteristics before and after deacidification and acid-doped viscosity: (a) viscosity changes of deacidified oil and acid components in toluene solution and (b) viscosity changes of deacidified oil mixed with different proportions of acid.

From the perspective of molecular weight distribution, the acidic components exhibited larger molecular weights with narrower distribution ranges, indicating a more uniform distribution of naphthenic acid. Conversely, the nonacid components exhibited lower molecular weights but wider distribution ranges, consisting mainly of small molecules with a small proportion of macromolecules. Thus, the deacidified oil contained macromolecular nitrogen-containing heteroatom compounds (Figure 4).

#### 3.3.2 Analysis of Viscosity Change after Deacidification

**Figure 5:**
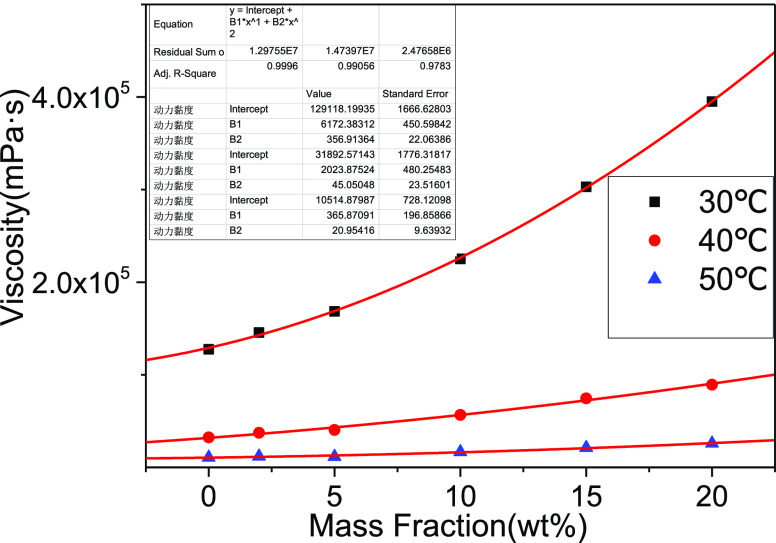
Change in viscosity of deacidified oil by the addition of acid components.

It can be seen from Figure 5 that the contribution of petroleum acid to viscosity is not linearly increasing, so according to the Arrhenius mixing rule, which is generally applicable in petroleum systems, the viscosity of mixed oil is
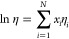
1The mixed system of acid and deacidified oil can be regarded as a binary system, and the concept of equivalent viscosity η_asp of petroleum acid is proposed
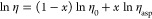
2
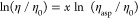
3
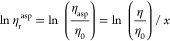
4
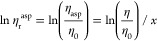
5
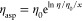
6

As can be seen in Table 3, the order of magnitude of the equivalent viscosity of the petroleum acid varies from the power of 10^4^ to the power of 10^7^, and from the point of view of the temperature, the equivalent viscosity is larger at a low temperature, indicating that the petroleum acid molecules are more likely to aggregate and less mobile at a low temperature.

**Table 3:** Equivalent Viscosity of the Acid Component in Deacidified Oil

equivalent viscosity (°C)	2 wt %	5 wt %	10 wt %	15 wt %	20 wt %
30	9.68 × 10^7^	3.36 × 10^7^	3.76 × 10^7^	4.09 × 10^7^	3.65 × 10^7^
40	4.20 × 10^7^	2.80 × 10^6^	8.83 × 10^6^	8.47 × 10^6^	5.21 × 10^6^
50	3.83 × 10^6^	5.93 × 10^4^	9.74 × 10^5^	1.05 × 10^6^	9.21 × 10^5^

**Figure 6:**
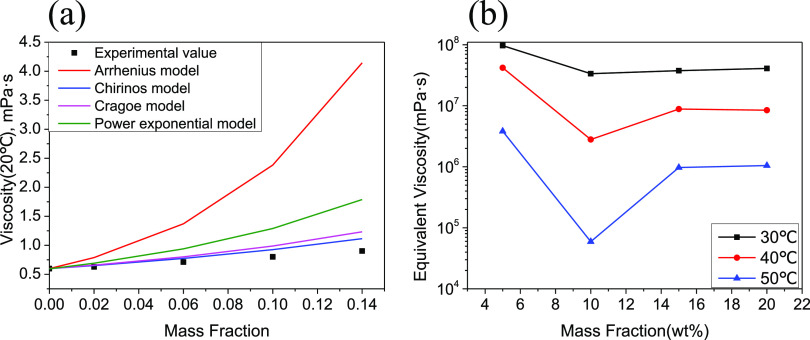
Changes in the viscosities of acid and deacidified oil: (a) viscosity–concentration relationship of nonacid in the solvent and (b) equivalent viscosity of the acid components at different temperatures (single logarithmic model).

As can be seen from Figure 6a and Table 4, according to the viscosity mixing model of deacidified oil in the toluene solvent, the chirinos model and the cragoe model have the smallest AARD%.

**Table 4:** Viscosity Tuning Model of Deacidified Oil in the Toluene Solvent

viscosity model	AARD%
Arrhenius model	135.03
Chirinos model	10.27
Cragoe model	15.43
power exponential model	40.37

As can be seen from Figure 6b, the equivalent viscosity has a downward trend between 2 and 10 wt %, indicating that hydrogen bond force may not be formed. The viscosity changes are small, and between 10 and 20 wt %, the equivalent viscosity gradually increases, indicating that the force or acid–base interaction increases.

In order to describe the relationship between viscosity and temperature at different acidity ratios, we have measured the viscosity of the blended deacidified oil with acidity fraction at each temperature point, and introduced Walther viscosity–temperature equation to fit the viscosity–temperature relationship of the blended oil sample. The equation is
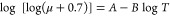
7

As can be seen from Table 5, the regression coefficient *R*^2^ of the viscosity–temperature curve of acid components in deacidified oil predicted by the Walther equation is above 0.99, and the AARD % is within 10. It can be considered that the Walther equation can better describe the viscosity–temperature properties of acid components in deacidified oil with different contents and can accurately predict the viscosity. It can also better describe the law of viscosity change with temperature.

**Table 5:** Changes in Viscosity–Temperature Parameters of Acid Components in Deacidified Oil

samples	*A*	*B*	*R*^2^	AARD%
Chunfeng heavy oil	9.4284	3.507	0.997	7.40
deacidified oil	9.9531	3.7255	0.9999	3.31
deacidified oil + 2 wt % acid component	9.5987	3.5816	0.9993	3.01
deacidified oil + 5 wt % acid component	10.138	3.7967	0.9991	4.64
deacidified oil + 10 wt % acid component	9.8183	3.663	0.9999	1.82
deacidified oil + 15 wt % acid component	9.8291	3.6632	0.9998	2.03
deacidified oil + 20 wt % acid component	9.8342	3.6619	0.9997	2.10

**Figure 7:**
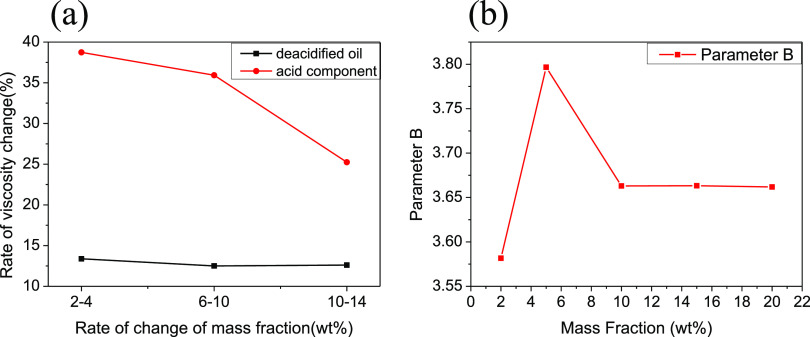
Viscosity change rate and viscosity–temperature parameter changes of acid components and deacidification oil: (a) change rate of deacidified oil and acid component in the toluene solvent and (b) changes in viscosity–temperature parameters of acid components in deacidified oil.

From Figure 7a, the viscosity change rate of the acid in the solvent is higher than that of the deacidified oil in the solvent. Additionally, Figure 4b demonstrates that at 30 °C, the viscosity of the deacidified oil phase decreases by 43.11% compared to that of Chunfeng heavy oil, indicating a significant contribution of petroleum acid to the viscosity of heavy oil. Figure 7a reveals that the double logarithmic model has the smallest error for the viscosity change of the deacidified oil in toluene solvent, suggesting its suitability as a viscosity blending model. Since the acid component exhibits a semisolid state at room temperature, its viscosity cannot be directly measured. However, the equivalent viscosity change of the acid component can be inferred from Figure 6b. The virtual viscosity demonstrates a trend of initially decreasing, followed by an increase and eventual leveling off. Figure 7b further illustrates that the viscosity–temperature parameter B, representing the slope, initially increases, then decreases, and finally becomes gentler within the mass fraction range of 2–20%.

### 3.4 Analysis of Molecular Composition Changes before and after Deacidification

**Figure 8:**
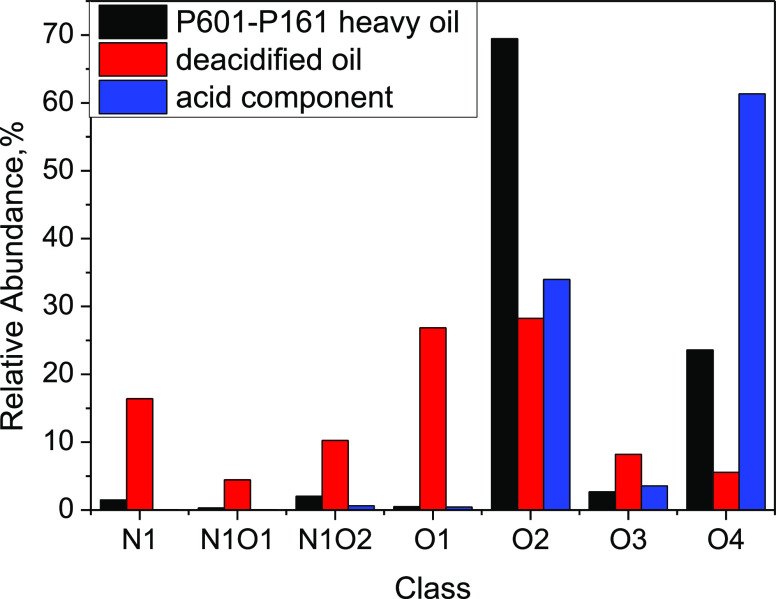
Distribution of heteroatom compounds in heavy oil and deacidified components.

Electrospray ionization (ESI), in combination with Fourier transform ion cyclotron resonance mass spectrometry (FT-ICR MS), is considered one of the most advanced methods for analyzing complex organic compounds with polarity in heavy petroleum products. Negative-ion ESI allows for selective ionization of trace acidic compounds present in a complex hydrocarbon matrix background. FT-ICR MS offers ultrahigh mass accuracy and resolution, enabling precise analysis of molecular composition. In the samples, negative-ion ESI detected petroleum acids (including carboxylic acids and phenolic compounds) as well as nonbasic nitrides (pyrrole nitrides). The mass spectrum peaks ranged between *m*/*z* 200 and 900, and the compounds exhibited continuous distribution. This continuous spectrum distribution indicates the highly complex composition of the compounds. Xinjiang heavy oil samples and the separated components were analyzed using FT-ICR MS, and the obtained spectra are presented in Figure 8.

According to Figure 8, after deacidification by alkali-modified silica gel chromatographic column, the O2 and O4 compounds in the crude oil are mainly enriched in the acidic fraction, and the contents of N1, O1, and O2 compounds in the deacidification oil is relatively high. It is mostly monoacid and diacid.

**Figure 9:**
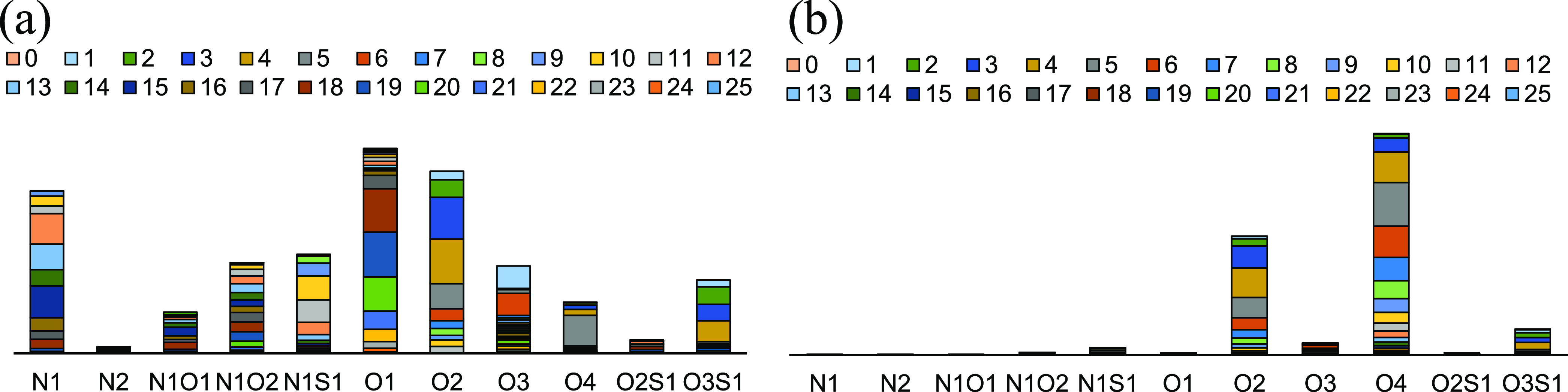
Distribution of heteroatom compounds in deacidified oil and acid component: (a) distribution of heteroatom compounds in deacidified oil and (b) distribution of heteroatom compounds in the acid component.

According to Figure 9, after deacidification by alkali-modified silica gel column, we found that both O2 and O4 compounds were enriched in the acidic fraction. Acidic compounds in crude oil can have a significant effect on viscosity; hence, to analyze the changes in chemical composition before and after deacidification, we studied the equivalent double bond number (DBE) of compounds such as O2 and O4, which is defined as the number of rings and double bonds containing carbon in a petrochemical component
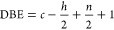
8

**Figure 10:**
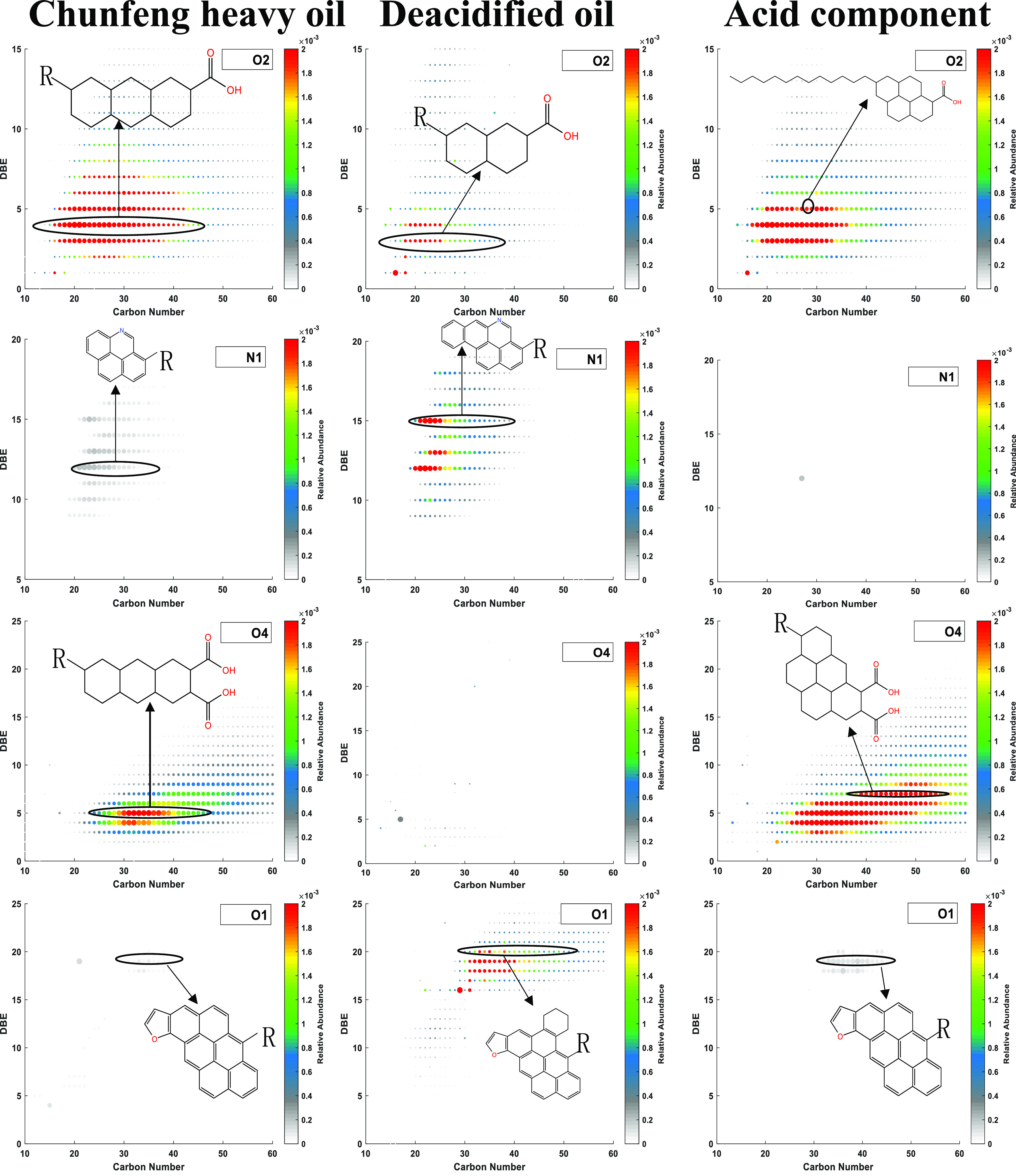
DBE–carbon number distribution of heavy oil and deacidified components.

According to Figure 10, Chunfeng heavy oil primarily lacks N1 compounds and is mainly composed of oxygen-containing compounds, with O2 and O4 compounds constituting the largest proportion. The distribution of O2 compounds is concentrated within the range of DBE 2–7 and carbon numbers 18–40. Similarly, the distribution of O4 compounds is concentrated within the range of DBE 4–5 and carbon numbers 30–40. In the deacidified oil, the O2 compounds decreased by 51% compared to crude oil. On the other hand, the distribution of N1 compounds was concentrated within the range of DBE 12–15 and carbon numbers 20–30, while the distribution of O2 compounds was concentrated within the range of DBE 3–5 and carbon numbers 18–35. Almost all acidic components are composed of O2 and O4 compounds.

Compared with crude oil, the DBE and carbon number distribution range of O2 compounds in deacidified oil are narrower. Compared with crude oil, the DBE and carbon number distribution range of O4 compounds in deacidified oil are wider, the carbon number is distributed in the range of 25–70, and the DBE is concentrated in the range of 2–10, indicating that this method can separate macromolecular diacids.

Considering the viscosity data, the removal of O2 and O4 compounds in the deacidified oil resulted in a 43.11% decrease in viscosity at 30 °C compared to crude oil, indicating that monoacid and diacid components greatly influenced the viscosity of heavy oil.

**Figure 11:**
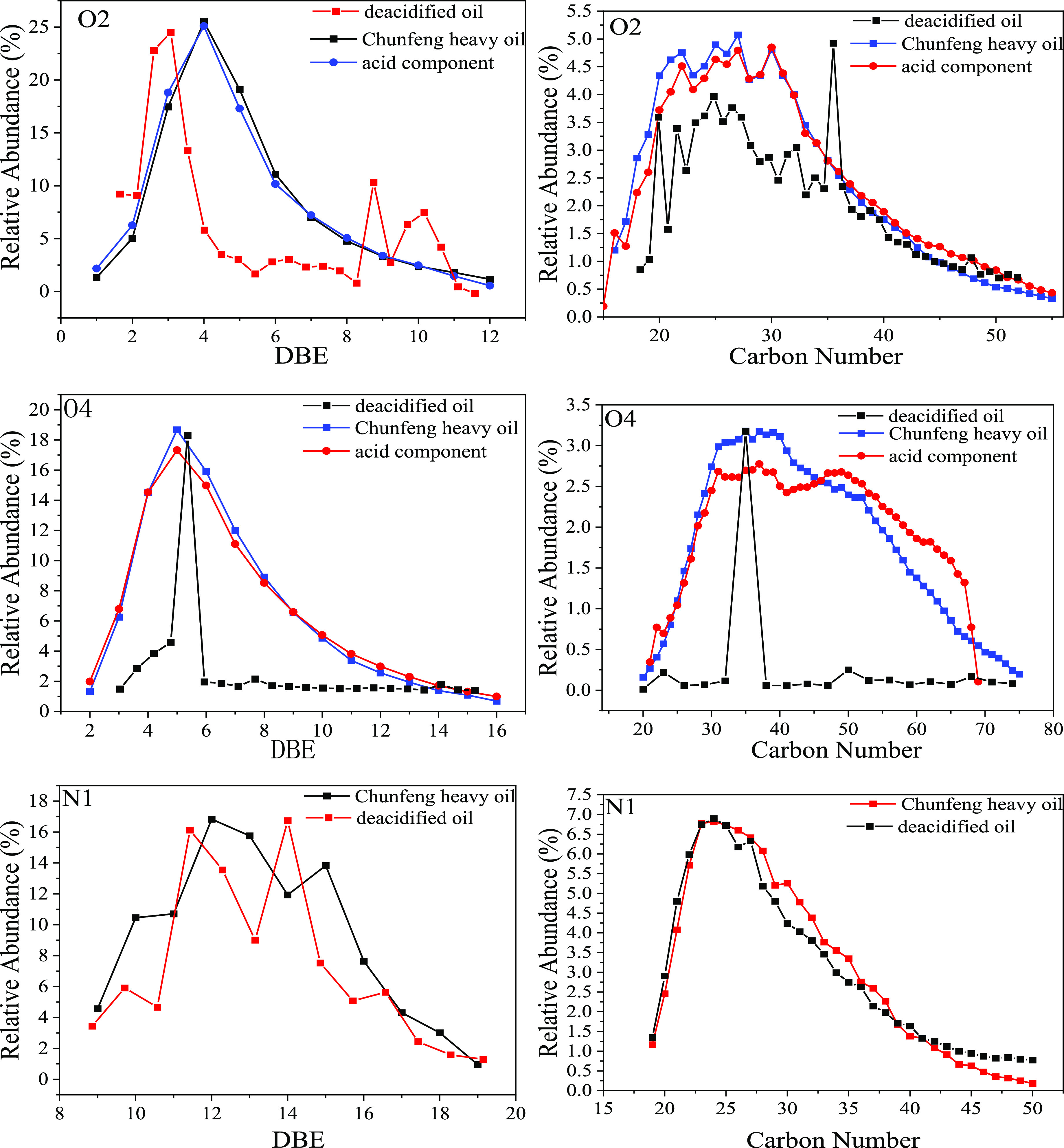
Comparison of DBE and carbon number distribution of heteroatomic compounds before and after deacidification.

As can be seen from Figure 11, the DBE distribution centers are all at DBE = 4, indicating that the O2 class compounds are mainly mono-acids with three cycloalkyl rings. The main molecular structures and contents are shown in Tables 6 and 7. The center of the carbon number distribution is around 28. The DBE distribution center of O4 compounds is DBE = 5, and the carbon number distribution center is around 40, indicating that the diacid is mainly composed of three cycloalkane rings. Although O2 and O4 compounds in deacidified oil also have distribution centers, their response degree is much lower than that of crude oil and acidic fractions, so their relative content is also low. This result can be verified from the change of acid value.

**Table 6:**
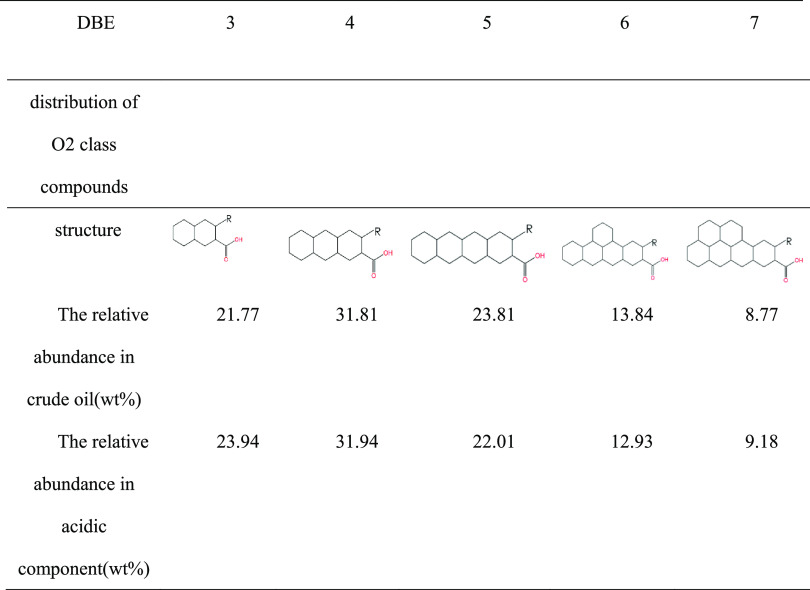
DBE Structure and Relative Abundance Distribution of O2 Class Compounds in Crude Oil and Acidic Components

**Table 7:**
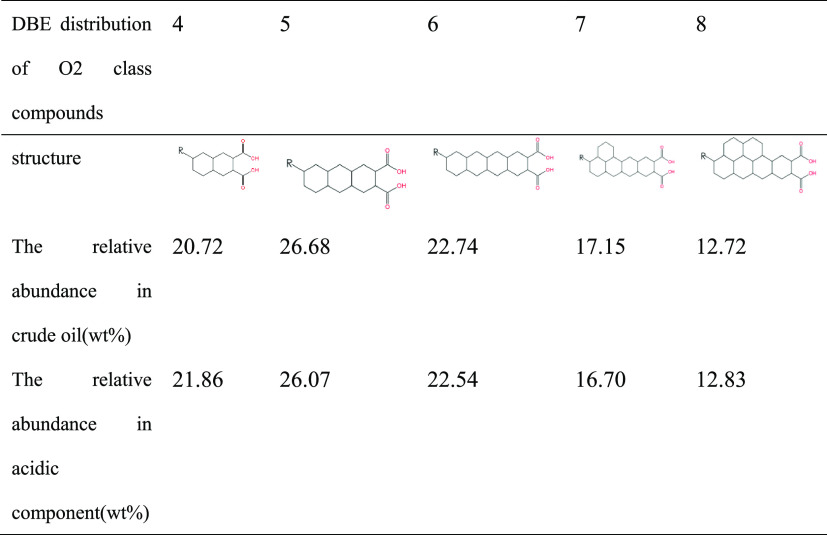
DBE Structure and Relative Abundance Distribution of O4 Class Compounds in Crude Oil and Acidic Components

## 4 Conclusions

Heavy oil with low asphaltene content may also have high viscosity. In this study, alkali-modified silica gel column chromatography was used to successfully separate the petroleum acid components, which were evaluated for their viscosities. The acid component shows much high viscosity than the deacidified oil. The deacidified oil exhibited a viscosity reduction of ∼40% after separation. After deacidification by the alkali-modified silica gel chromatographic column, the O2 and O4 compounds in the crude oil are mainly enriched in the acidic fraction, and the contents of N1, O1, and O2 compounds in the deacidification oil are relatively high. The acid component primarily consisted of O2 and O4 compounds, indicating the enrichment of monocyclic naphthenic acid and bicyclic naphthenic acid. Thus, the effects of different structures and contents of petroleum acid on the viscosity of heavy oil should be further investigated.

The acidic component can enhance hydrogen bonds and acid–base interactions, making it easier for molecules to polymerize, thereby increasing viscosity. From the effect of the acid content on the viscosity of the deacidified oil, it can be seen that the aggregates formed were larger and less mobile when the proportion of added petroleum acid was higher. Deacidification can affect the interaction between substances in crude oil and change its internal micelle structure. It also leads to changes in the structural stability of asphaltene/colloid micelles, which can affect the viscosity of crude oil. Since the composition and physical structure of crude oil are too complex, the viscosity is not caused by a single component. It is the result of a combination of various components and their interactions. The effect of deacidification on the interaction between crude oil components is complex and requires further investigation. Future work will investigate the interaction between naphthenic acids and other substances both experimentally and by simulation.

## References

[ref1] Luo P.; Gu Y. Effects of asphaltene content on the heavy oil viscosity at different temperatures. Fuel 2007, 86, 1069–1078. 10.1016/j.fuel.2006.10.017.

[ref2] Thomas S. Enhanced oil recovery-an overview. Oil Gas Sci. Technol.-Rev. de l’IFP 2008, 63, 9–19. 10.2516/ogst:2007060.

[ref3] Wu Z.; Liu H.; Pang Z.; Wu C.; Gao M. Pore-scale experiment on blocking characteristics and EOR mechanisms of nitrogen foam for heavy oil: a 2D visualized study. Energy Fuels 2016, 30, 9106–9113. 10.1021/acs.energyfuels.6b01769.

[ref4] Wu Z.; Liu H.; Xue W. 3D experimental investigation on enhanced oil recovery by flue gas coupled with steam in thick oil reservoirs. Energy Fuels 2018, 32, 279–286. 10.1021/acs.energyfuels.7b03081.

[ref5] Yang C.-z.; Han D.-k. Present status of EOR in the Chinese petroleum industry and its future. J. Pet. Sci. Eng. 1991, 6, 175–189. 10.1016/0920-4105(91)90036-M.

[ref6] Yigang L.; Jian Z.; Hua Z.; Weihang Z.; Fayuan Z.; Qiuxia W.; Shanshan L. Improvement and application of high efficiency water supply system for heavy oil thermal recovery. Sci. Technol. Eng. 2019, 19, 211–214.

[ref7] Chunhao W.; Ruihe W.; Weidong Z.; Luopeng L. Design of experiment system and experimental study in heavy oil viscosity reduction with cavitation jet. Sci. Technol. Eng. 2019, 19, 74–79.

[ref8] Guo J.; Yang Y.; Zhang D.; Wu W.; Yang Z.; He L. A general model for predicting apparent viscosity of crude oil or emulsion in laminar pipeline at high pressures. J. Pet. Sci. Eng. 2018, 160, 12–23. 10.1016/j.petrol.2017.10.034.

[ref9] Ghanavati M.; Shojaei M.-J.; SA A. R. Effects of asphaltene content and temperature on viscosity of Iranian heavy crude oil: experimental and modeling study. Energy Fuels 2013, 27, 7217–7232. 10.1021/ef400776h.

[ref10] Argillier J.; Coustet C.; Henaut I. Heavy Oil Rheology as a Function of Asphaltene and Resin Content and Temperature; SPE International Thermal Operations and Heavy Oil Symposium, SPE, 2002; p SPE-79496-MS.

[ref11] Li X.; Chi P.; Guo X.; Sun Q. Effects of asphaltene concentration and asphaltene agglomeration on viscosity. Fuel 2019, 255, 115825 10.1016/j.fuel.2019.115825.

[ref12] Moud A. A. Asphaltene induced changes in rheological properties: A review. Fuel 2022, 316, 123372 10.1016/j.fuel.2022.123372.

[ref13] Sheu E. Y.; Mullins O. C. Fundamentals and applications; Springer, 1995.

[ref14] Chilingarian G.; Yen T. Asphaltenes and Asphalts, 1; Elsevier, 1994.

[ref15] Mack C. Colloid chemistry of asphalts. J. Phys. Chem. A 1932, 36, 2901–2914. 10.1021/j150342a005.

[ref16] Dealy J. M. Rheological properties of oil sand bitumens. Can. J. Chem. Eng. 1979, 57, 677–683. 10.1002/cjce.5450570604.

[ref17] Mansour E.; Farag A.; El-Dars F.; Desouky S.; Batanoni M.; Mahmoud M. Predicting PVT properties of Egyptian crude oils by a modified Soave–Redlich–Kowng equation of state. Egypt. J. Pet. 2013, 22, 137–148. 10.1016/j.ejpe.2012.09.005.

[ref18] Mansour E.; Desouky S.; El Aily M.; Helmi M. The effect of asphaltene content on predicting heavy dead oils viscosity: Experimental and modeling study. Fuel 2018, 212, 405–411. 10.1016/j.fuel.2017.10.024.

[ref19] Sirota E. B.; Lin M. Y. Physical behavior of asphaltenes. Energy Fuels 2007, 21, 2809–2815. 10.1021/ef060634c.

[ref20] Hasan M. A.; Shaw J. M. Rheology of reconstituted crude oils: Artifacts and asphaltenes. Energy Fuels 2010, 24, 6417–6427. 10.1021/ef101185x.

[ref21] Gao S.; Moran K.; Xu Z.; Masliyah J. Role of naphthenic acids in stabilizing water-in-diluted model oil emulsions. J. Phys. Chem. B 2010, 114, 7710–7718. 10.1021/jp910855q. 20496916

[ref22] Bertheussen A.; Simon Sb.; Sjöblom J. Equilibrium partitioning of naphthenic acid mixture, part 1: commercial naphthenic acid mixture. Energy Fuels 2018, 32, 7519–7538. 10.1021/acs.energyfuels.8b01494.

[ref23] Bertheussen A.; Simon Sb.; Sjöblom J. Equilibrium partitioning of naphthenic acid mixture Part 2: crude oil-extracted naphthenic acids. Energy Fuels 2018, 32, 9142–9158. 10.1021/acs.energyfuels.8b01870.

[ref24] Purcell J. M.; Hendrickson C. L.; Rodgers R. P.; Marshall A. G. Atmospheric pressure photoionization Fourier transform ion cyclotron resonance mass spectrometry for complex mixture analysis. Anal. Chem. 2006, 78, 5906–5912. 10.1021/ac060754h. 16906739

[ref25] Pu W.; He M.; Yang X.; Liu R.; Shen C. Experimental study on the key influencing factors of phase inversion and stability of heavy oil emulsion: Asphaltene, resin and petroleum acid. Fuel 2022, 311, 122631 10.1016/j.fuel.2021.122631.

[ref26] Yang X.; Czarnecki J. Tracing sodium naphthenate in asphaltenes precipitated from athabasca bitumen. Energy Fuels 2005, 19, 2455–2459. 10.1021/ef058017w.

[ref27] Seifert W. K.; Howells W. G. Interfacially active acids in a California crude oil. Isolation of carboxylic acids and phenols. Anal. Chem. 1969, 41, 554–562. 10.1021/ac60273a002.

[ref28] Colati K. A.; Dalmaschio G. P.; de Castro E. V.; Gomes A. O.; Vaz B. G.; Romão W. Monitoring the liquid/liquid extraction of naphthenic acids in brazilian crude oil using electrospray ionization FT-ICR mass spectrometry (ESI FT-ICR MS). Fuel 2013, 108, 647–655. 10.1016/j.fuel.2013.02.007.

[ref29] Jones D. M.; Watson J.; Meredith W.; Chen M.; Bennett B. Determination of naphthenic acids in crude oils using nonaqueous ion exchange solid-phase extraction. Anal. Chem. 2001, 73, 703–707. 10.1021/ac000621a. 11217788

[ref30] Ni W.; Zhu G.; Liu F.; Li Z.; Xie C.; Han Y. Carboxylic acids in petroleum: Separation, analysis, and geochemical significance. Energy Fuels 2021, 35, 12828–12844. 10.1021/acs.energyfuels.1c01518.

[ref31] Shah S. N.; Lethesh K. C.; Abdul Mutalib M.; Mohd Pilus R. B. Extraction and recovery of naphthenic acid from acidic oil using supported ionic liquid phases (SILPs). Chem. Prod. Process Model. 2015, 10, 221–228. 10.1515/cppm-2014-0036.

[ref32] Barakat A. O.; Peakman T. M.; Rullkötter J. Isolation and structural characterization of 10-oxo-octadecanoic acid in some lacustrine sediments from the Nördlinger Ries (southern Germany). Org. Geochem. 1994, 21, 841–847. 10.1016/0146-6380(94)90043-4.

[ref33] Qian K. Molecular characterization of heavy petroleum by mass spectrometry and related techniques. Energy Fuels 2021, 35, 18008–18018. 10.1021/acs.energyfuels.1c01783.

[ref34] Barrow M. P.; Peru K. M.; McMartin D. W.; Headley J. V. Effects of extraction pH on the Fourier transform ion cyclotron resonance mass spectrometry profiles of Athabasca oil sands process water. Energy Fuels 2016, 30, 3615–3621. 10.1021/acs.energyfuels.5b02086.

[ref35] Li M.; Cheng D.; Pan X.; Dou L.; Hou D.; Shi Q.; Wen Z.; Tang Y.; Achal S.; Milovic M.; Tremblay L. Characterization of petroleum acids using combined FT-IR, FT-ICR–MS and GC–MS: Implications for the origin of high acidity oils in the Muglad Basin, Sudan. Org. Geochem. 2010, 41, 959–965. 10.1016/j.orggeochem.2010.03.006.

[ref36] Chi Y.; Liu B.; Chen Y.; Wang B.; Bai L.; Wang L.; Xu Y. J. U. R. Geochemical characteristics and mechanisms of densification of pre-Jurassic crude oil in the Tainan Sag, Turpan-Hami Basin, China. Unconv. Resour. 2022, 2, 183–191. 10.1016/j.uncres.2022.10.003.

[ref37] Liu B.; Huang Z.; Tu X.; Sang T.; Chen X. J. P. S. Oil accumulation related to migration of source kitchens in the Lukeqin structural belt, Turpan-Hami Basin, China. Petroleum Science 2010, 7, 355–361. 10.1007/s12182-010-0077-x.

[ref38] Zhi-huan Z.; Hong-jun L.; Wei L.; Jia-jia F.; Kui X.; Li-ming Q.; Wei-jun X.; Lei Z. Environment, Origin and Accumulation Process of Heavy Oil in Chepaizi Area of Junggar Basin. J. Earth Sci. Environ. 2014, 36, 18–32.

